# Large animal models in gynecology: status and future perspectives

**DOI:** 10.3389/fvets.2025.1588098

**Published:** 2025-05-07

**Authors:** Dan Zhao, Xue Li, Xu Zheng, Xiangrui Xie, Yanan Zhao, Yang Liu

**Affiliations:** ^1^College of Acupuncture and Massage, Changchun University of Chinese Medicine, Changchun, Jilin, China; ^2^Department of Gynecology, The Third Affiliated Hospital of Changchun University of Chinese Medicine, Changchun, Jilin, China; ^3^Department of Tuina, The First Affiliated Hospital of Henan University of Traditional Chinese Medicine, Zhengzhou, Henan, China; ^4^Institute of Acupuncture and Moxibustion, China Academy of Chinese Medical Sciences, Beijing, China; ^5^Department of Acupuncture, Affiliated Hospital of Changchun University of Chinese Medicine, Changchun, Jilin, China

**Keywords:** large animal models, gynecological diseases, non-human primates, pigs, sheep

## Abstract

The purpose of this review is to evaluate the effectiveness of large animal models in gynecology research and provide future perspectives. Gynecological diseases are diverse and pose a serious threat to women’s physical and mental health. In addition to the commonly used small animal models, large animal models have gradually entered the field of gynecological research. Results suggest that large animal models offer significant advantages in simulating human physiological processes, despite ethical and practical challenges. This paper reviews the application of large animal models in the study of gynecological diseases, provides a summary of the research characteristics of large animal models, analyses the advantages and challenges of these models in disease research, and compares the research differences between large and small animal models. It also discusses the relationship between these models and new alternative models, with a view to providing more new ideas for the selection of animal models in the study of gynecological diseases.

## 1 Introduction

As society develops and the pressures of modern life increase, the number of female diseases derived from a lack of education, sexual violence, lack of contraception and psychological problems is rising (1). According to recent studies, it is estimated that around 1 in 4 women suffer from gynecological disorders, with menstrual disorders, infertility, and gynecological cancers being the most prevalent (2). These conditions not only cause significant physical pain but also contribute to emotional distress, reduced quality of life, and long-term mental health challenges. For example, approximately 10–15% of women globally suffer from endometriosis, and nearly 30% of couples experience infertility, affecting both physical and mental health (3). These ailments present challenges due to their high prevalence, intricate pathophysiological mechanisms, and the critical need for early detection and treatment in medical research (4). It is therefore imperative that experimental models are developed to simulate human physiological processes in order to conduct more research, explore disease mechanisms, and develop novel medical diagnostics and treatments. However, animal models are selected not only for their ability to simulate human physiology but also for ethical considerations, availability, cost-effectiveness, and their relevance to specific research questions. These factors, including the feasibility of model selection and the alignment with research objectives, play a crucial role in determining the appropriate model for studying complex diseases. New alternative models, such as organ-on-a-chip systems and computational models, have been introduced to enhance the selection of animal models for gynecological diseases. These models allow for more ethical, efficient, and accurate simulations of human physiological processes, providing novel insights into disease mechanisms, drug testing, and patient-specific therapies. Such models are gaining recognition for their potential to complement traditional animal models and reduce reliance on animal use, addressing both scientific and ethical considerations.

**Figure 1:**
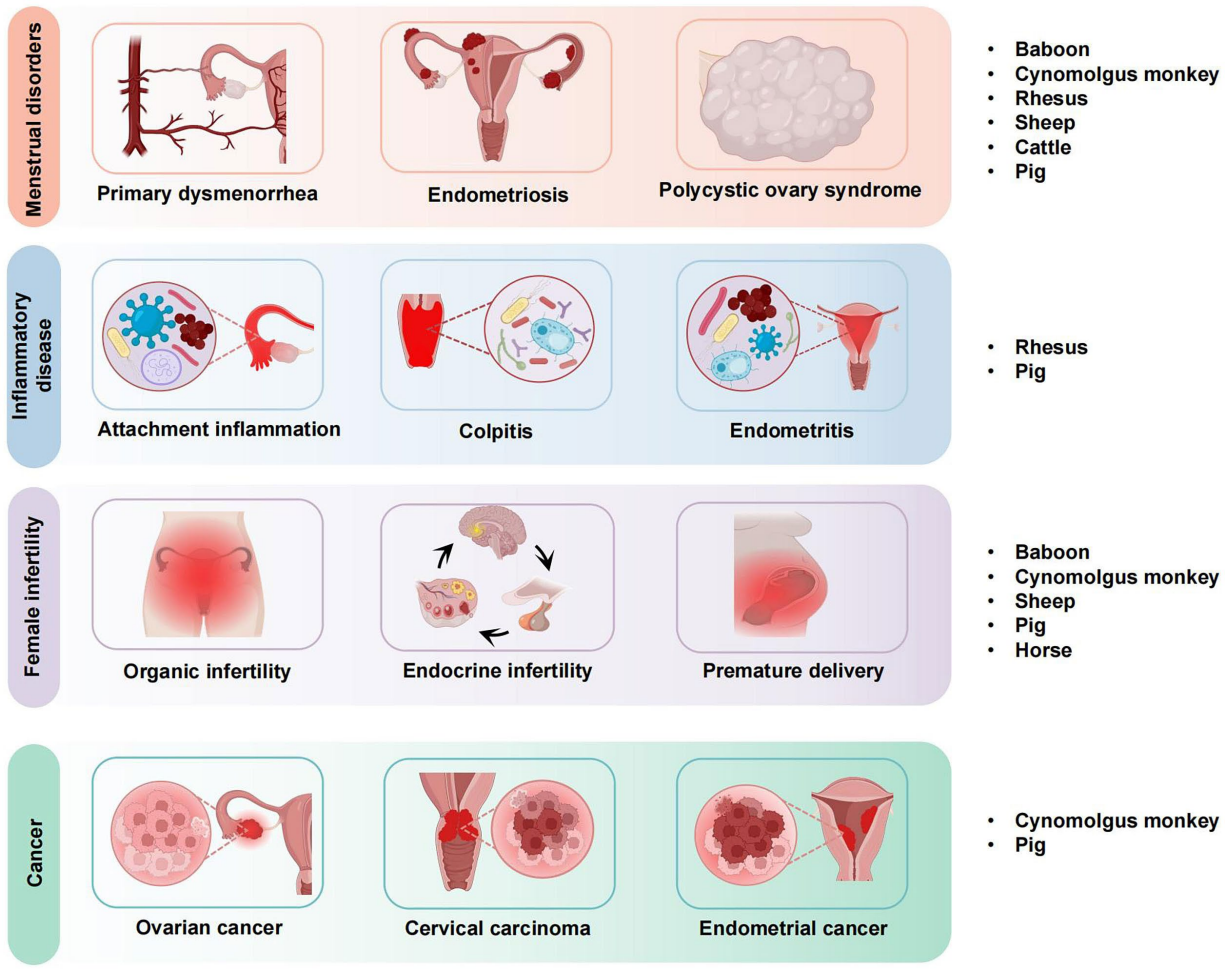
Application of large animal models in gynecological diseases.

Large animal models, such as pigs, sheep, and non-human primates, are crucial in gynecologic research due to their physiological similarities to humans. Unlike small animal models, which often lack the complexity of human reproductive systems, large animals exhibit more accurate representations of human physiology, including hormonal regulation, organ structure, and response to disease (5). For example, pigs have a reproductive cycle similar to that of humans, and non-human primates share many genetic and anatomical traits with humans, making them valuable models for studying menstrual disorders, infertility, and cancer (6). For instance, a 2021 report by Makowska (7) indicated that rodents, particularly mice and rats, account for over 70% of laboratory animal use globally. Additionally, studies by Baker and Lind (8, 9) have highlighted the growing use of non-human primates, pigs, and other large animals in specific research fields such as reproductive biology and disease modeling. Of these, rodents, notably mice and rats, have historically dominated the market share, accounting for over 70% of laboratory animal use (10). Moreover, in recent years, the development of additional animal models and the occurrence of epidemics have led to a notable increase in the utilization of experimental large animals (11). The data indicate that in 2021, the market share of non-human primates surpassed that of larger strains of laboratory mice for the first time in China, with a demand for experimental non-primate animals, including pigs, reaching 129,200. Additionally, the annual demand for experimental non-primate animals reached 129,200, while the demand for other large animal models, such as experimental pigs, was 66,000. Currently, the primary citation is based on a study involving Chinese women aged 50–70 years. However, to broaden the scope of these findings, additional studies encompassing diverse populations and age groups, both in China and globally, should be cited. For example, studies involving younger women or those from different ethnic backgrounds could provide more comprehensive insights into the topic. Overall, the quantity scale of these animals was approximately equal to that of non-human primates (12). It is evident that in the domain of scientific research and medicine, large animal models, which exhibit considerable similarities to the human body in a multitude of aspects, including genetics, physiology, and structure, have garnered significant attention from researchers, particularly in the fields of reproduction, dermatology, immunology, and metabolism, where they offer distinct advantages. These models offer distinct advantages, such as more accurate simulations of human physiological processes, which is critical for studies in reproductive biology, disease modeling, and drug testing (13). These advantages make them particularly valuable in preclinical trials and in modeling complex human diseases that require a more detailed biological context. This review introduces new ideas for the selection of animal models in the study of gynecological diseases. It presents the application of large animal models in the study of common gynecological diseases (see Figure 1), analyses the current status of their research, clarifies their advantages and challenges in the study of this field, and explores the value of applying large animal models to the preclinical experimental study of gynecological diseases and the significance of their use in the preclinical experimental study of gynecological diseases.

## 2 Large animal models and gynecological diseases

Large animal models play an important role in gynecologic research due to their physiologic similarity to humans, allowing for more accurate studies of disease and therapy. These models help bridge the gap between small animal models and human clinical trials, enabling better understanding of disease mechanisms and the testing of therapeutic interventions. Methods to create animal models of gynecological diseases include spontaneous disease models, induced disease models, and genetically modified models, which simulate human diseases such as endometriosis, polycystic ovary syndrome, and uterine cancer. Large animal models are a valuable tool in the study of gynecological diseases, particularly in the investigation of pathogenesis, the evaluation of diagnostic techniques and the creation of innovative therapies or drugs (14). Large animal models are important in the study of common gynecological diseases, offering distinct advantages in simulating human conditions and advancing diagnostic and therapeutic research (15). At present, researchers have the option of utilizing a variety of large animal models, employing different modeling techniques, including spontaneous models, induced models, transplanted models and genetic intervention models. As illustrated in Table 1, the pathogenesis and progression of gynecological diseases were modeled to facilitate the generation of novel diagnostic and therapeutic strategies for clinical applications.

**Table 1:** Application of large animal models.

Animal models	Scope of application	Advantages	Limitations
Baboon	1. Drug development, pharmacodynamic and toxicological studies. 2. Spontaneous/ induced endometriosis models.	1. The menstrual cycle is similar to that of females. 2. Developmental homology with humans. 3. The spontaneous model is non-invasive.	1. Higher feeding and training requirements. 2. Medication and food are more expensive (62). 3. Ethical issues are more important (55).
Cynomolgus monkey	1. An investigation into the pathological mechanisms of endometriosis (26). 2. An evaluation of the potential for autologous uterine transplantation (147).	The menstrual manifestations are readily apparent and may be spontaneously molded.	Endangered species.
Rhesus monkey	1. The genetic epidemiology of endometriosis and the dose-effect relationship of drugs will be examined (34). 2. The etiology and genetic variants of polycystic ovary syndrome (148). 3. The mechanisms of infection and the analysis of bacterial flora in inflammatory diseases of the reproductive tract (43).	1. Analogous to human genetic material, capable of facile molding. 2. Harbor a diverse array of human microbial flora.	Higher price and limited experimentation.
Sheep	1. An investigation of the hormonal influences on uterine development (149). 2. Models for the induction of polycystic ovary syndrome (150). 3. The objective is to measure and analyze the development of the placenta, the dynamics of follicles and the activity of neurotransmitters (151). 4. Autologous uterine transplantation (152).	1. The development of reproductive organs in this species is analogous to that observed in the human body. 2. The reproductive trajectory is analogous to that observed in females (153). 3. The vascular development and nutritional exchange of the placenta exhibits similarities to that of the female.	1. Have disparate gestation periods (154). 2. The genitalia are structurally dissimilar to those of the human body (67).
Pig	1. Non-invasive surgical procedures for the treatment of adenomyosis (155). 2. Autologous uterus transplantation (156). 3. Investigation of the etiology of gynecological inflammation and the development of novel pharmaceutical agents (48).	1. The feasibility of autograft vascular anastomosis was confirmed (65). 2. The anatomy and size of the abdomen are similar to those of the human body (157).	The study was conducted with a relatively small sample size and without a comprehensive risk assessment.
Cow	1. The observation of sinusoidal follicles in motion (158). 2. The application of cryocooling techniques to extend the viability of follicles (159).	1. The time points in the estrous cycle are analogous to those observed in the female menstrual cycle. 2. It establishes the foundation for the mechanism of the female follicular wave pattern.	The level of follicle-stimulating hormone (FSH) present at the time of ovulation varies between women (160).
Horse	1. Development of antimicrobial agents (161). 2. Mechanism excavation of low ovarian reserve function (162).	1. The follicles are of a considerable size, thus facilitating observation. 2. The follicular recruitment waves and dominant follicles are analogous to those observed in women (68).	The position of the ovaries differs from that observed in women (163).

### 2.1 Menstrual disorders

The use of animal models, particularly non-human primates, in the study of menstrual disorders dates to the early 20th century (16). Researchers first observed menstrual-like cycles in non-human primates, which paved the way for understanding the hormonal regulation of menstruation (17, 18). These models have provided invaluable insights into disorders such as endometriosis, polycystic ovary syndrome, and amenorrhea, which were initially characterized and studied through the behavior and physiological changes observed in these animals. Since the International Federation of Gynecology and Obstetrics (FIGO) Working Group on Menstrual Disorders commenced efforts to establish a global consensus in 2005, the subject of women’s menstrual health has begun to attract significant attention from a range of stakeholders (19). Menstruation is a symbol of female fertility and an integral component of women’s health. The fundamental processes of menstruation are regulated by the hormones estrogen and progesterone, which induce cyclic necrosis and exfoliation of the endometrium (20). For example, a study involving rhesus monkeys demonstrated that endometriosis lesions could be induced and observed, with findings similar to those seen in human patients (21). These animal models have helped identify potential biomarkers for early diagnosis and have been used to evaluate the effectiveness of various therapeutic approaches, such as hormonal treatments and surgical interventions. However, the menstrual cycle in rhesus monkeys is most prominently observed in November and December, although it occurs throughout the year (22). Consequently, when selecting animal models for such diseases, researchers have prioritized those that exhibit similarities to female pelvic anatomy and reproductive characteristics.

As demonstrated in Table 2, menstruation was spontaneously initiated in all large animal models, and the estrous cycles exhibited notable similarities. Among these, the reproductive organs of non-human primates are most similar to those of humans. Non-human primates are frequently employed in research pertaining to the diagnosis and treatment of menstrual disorders, including endometriosis, adenomyosis, polycystic ovary syndrome, functional uterine bleeding, and other menstrual disorders. As early as 2007, researchers observed the location of pelvic lesions laparoscopically in non-human primates with endometriosis and found them to be highly comparable to those observed in humans. The baboon is a large non-human primate that allows for repetitive blood sampling and complex surgical operations when used as an animal model (23). It is genetic and protein expression similarity to human’s results in symptomatic manifestations akin to those found in female, evident at both macroscopic and microscopic levels. Additionally, the baboon’s short and straight cervix enables direct access to the uterine cavity for procedures such as endometrial biopsy, embryo transfer, pre-implantation embryo flushing, and hysteroscopy (24). This unique anatomical feature makes the baboon an ideal model for studying endometriosis, offering a non-invasive alternative to conventional invasive procedures. Numerous studies support the baboon as the most suitable and effective experimental animal model for investigating endometriosis (25).

**Table 2:** Menstrual characteristics of large animal models.

Animal models	Oestrus cycle (days)	Seasonality (N/A)	Sexual maturity	Menstruation	Uterus	Ovary
Baboon (164)	30–33	N	4–6 years old	Spontaneity	Single uterus	—
Cynomolgus monkey (165)	26–28	N	3–4 years old	Spontaneity	Single uterus	Size: 2 × 2.4 cm. Weight: 0.4–1 g.
Rhesus monkey (166)	23–33	A	2.5–3 years old	Spontaneity	Single uterus	Size: 1.1 × 1 cm. Weight: 0.4 g.
Sheep (53)	14–21	A	5–10 months	Spontaneity	Bicornuate uterus	Size: 1.7 × 1 cm. Weight: 1.35 g.
Pig (167)	18–23	N	5–6 months	Spontaneity	Bicornuate uterus	Size: 5 × 3 cm. Weight: 7–9 g.
Cow (162)	18–24	N	6–12 months	Spontaneity	Single uterus	Size: 2.5 × 2 cm. Weight: 15–20 g.
Horse (158)	19–22	A	10–18 months	Spontaneity	Single uterus	Size: 7.5 × 3 cm. Weight: 25–40 g.

N, Not Available; A, Available.

Nishimoto-Kakiuchi and colleagues discovered that adolescent cynomolgus monkeys between the ages of 11 to 20 exhibited heightened menstrual bleeding frequency and robust ovarian activity (26). These monkeys, due to their manageable size and ease of control in experimental settings, offer a more viable option for observing menstrual disease dynamics in comparison to human subjects. However, due to the fact that cynomolgus monkeys are an endangered species and resources are scarce, it has not been feasible to conduct experimental studies with a large data set (27). However, rhesus monkeys from India exhibit a highly regular menstrual cycle, occurring once per month (28). This regularity is mirrored in the human body in numerous ways, including genomics, anatomy and physiology, neurological function, and aging. As a result, rhesus monkeys present a more suitable model for exploring gynecological diseases, particularly in investigating gene function, structure, and epigenetic factors. In populations exhibiting a significant familial predisposition to polycystic ovary syndrome, there exists a minimum of 26 risk genes that govern diverse reproductive functions (29). Rhesus monkeys exhibit similarities to humans in aspects such as physiology, aging, and disease susceptibility (30). Consequently, researchers frequently select rhesus monkeys for the investigation of variant genes and dysfunction in polycystic ovary syndrome, with the objective of identifying potential therapeutic interventions. Moreover, investigations have revealed a 43% incidence of spontaneous endometriosis via laparoscopy at breeding sites of rhesus monkeys (31). Notably, an analysis of familial aggregation of the disease indicated a significantly elevated mean kinship coefficient for endometriosis among rhesus monkeys possessing complete genealogical data compared to unaffected kinship coefficients in a random sample (32). This has spurred the utilization of rhesus monkeys in genetic epidemiology studies on endometriosis, histopathology, and the assessment of drug dosage efficacy (33). It is worth mentioning that both cynomolgus monkeys and rhesus monkeys are classified under macaque monkeys, with the estrus cycle of macaque monkeys displaying distinct seasonality primarily in November and December (34). It is therefore imperative that researchers pay close attention to the specific characteristics of the estrous cycle of rhesus monkeys when selecting them for studies into menstrual diseases.

In addition to non-human primates, a number of large animals, including pigs, sheep and cattle, have contributed to the study of menstrual disorders. For instance, in the context of studying polycystic ovary syndrome through prenatal androgen investigations, manipulating testosterone levels in sheep during the fetal stage has led to the discovery of significant impacts on neuroendocrine feedback mechanisms in adulthood. This disruption has been linked to heightened pituitary response to gonadotropin-releasing hormone (GnRH), elevated luteinizing hormone levels, increased presence of functional androgens, and the development of a multifollicular ovarian morphology (35). Notably, these findings have also been associated with the initiation of preterm labor. Such insights derived from animal models present promising avenues for identifying novel therapeutic targets for polycystic ovary syndrome. In addition, lesions of the ovary are characterized mainly by granulosa cell and oocyte apoptosis, follicular atresia, decreased oocyte quality and embryonic developmental potential, oxidative stress and mitochondrial abnormalities, which ultimately lead to reduced fertility in women (36). Additionally, the physiological characteristics of large animal models with single follicles and multiple cycles are similar to those of women (37). When exploring the fluctuation patterns of follicular dynamics, cows are also often selected as a model to replace human subjects for real-time ultrasonography tests and to analyze the specific waveform characterization of their follicular dynamics, which facilitates the dissection of systemic dysfunctions of ovarian function in humans (38). Pigs, due to their comparable organ size to humans and consistent estrus cycle, have been utilized in magnetic resonance imaging-guided focused ultrasound surgical simulations of adenomyosis. These simulations involve the autologous endometrial implantation technique, circumventing issues related to extended drug-induced cycles and hormonal imbalances. This approach better reflects the characteristics of adenomyosis in the uterus, with a natural affinity to uterine tissue.

The utilization of large animal models in the investigation of menstrual disorders contributes significantly to advancing knowledge on the physiological intricacies of the female reproductive cycle and ovarian processes. Moreover, it offers valuable insights into the malfunctions affecting the uterus, ovaries, and associated reproductive organs, thereby enabling researchers to delve into innovative approaches for diagnosis and treatment.

### 2.2 Inflammatory diseases

In recent times, there has been a rise in gynecological inflammatory diseases linked to sexual transmission due to increased sexual openness, indicating a trend toward a younger affected demographic (39). Among these, pelvic inflammatory disease, which is caused by bacterial and viral infections, is a highly inflammatory response in the upper reproductive tract of women (including the endometrium, fallopian tubes and ovaries, etc.) (40). The chronic inflammatory state with an immune response often induces the emergence of ovarian cancer. Furthermore, mycoplasma infection-induced inflammation of the reproductive tract frequently results in significant complications, including infertility and preterm delivery, due to its prolonged nature, lasting for months to years (41). It is evident that gynecological inflammation should not be trivialized and may result in further complications if left untreated for an extended period.

The investigation of microbial communities in bacterial pelvic inflammatory disease and vaginitis has presented challenges in conducting analogous experiments in Lactobacillus-dominated mammals (42). A thorough examination utilizing 16SrRNA amplicons and shotgun metagenomic sequencing in rhesus monkeys revealed similarities in physiology and genetic makeup with female counterparts, along with a diverse array of vaginal flora shared with female microbiota. This discovery positions female rhesus monkeys as promising subjects for further research in this domain (43). Additionally, the utilization of non-human primates in the study of salpingitis through animal models is hindered by factors such as high costs, logistical difficulties, and ethical considerations, limiting their applicability (44). Researchers have noted that pigs are a valuable animal model due to their comparable anatomy and abdominal size to humans, making them suitable for simulating clinical conditions in research (45). Pigs have been successfully utilized in modeling endometritis by inducing inflammatory responses and vasodilation in the uterine horns using *Escherichia coli*, thus affecting the noradrenergic neurons in the caudal mesentery (46). This model has provided a solid foundation for the study of anti-inflammatory drugs. Pigs, particularly when employing endoscopic methods, offer researchers a clearer view of uterine lesions. Moreover, pigs are suitable for pharmacokinetic investigations due to their anisotropic growth patterns, a departure from human applications (47). In a study aimed at developing a novel formulation of the non-steroidal anti-inflammatory drug carprofen, an alternative to individual pig analysis was explored (48). This is also applicable to men, where similar genetic and physiological factors play a role. The study administered carprofen through both intravenous and intramuscular routes to assess the bioavailability of plasma samples pre-and post-intervention. The results revealed that using pigs as an animal model for such experiments not only did not compromise the concurrent analysis of pharmacokinetic profiles in the dataset but also conferred an advantage in data mining due to the longer half-life of pigs compared to humans. Pigs have a longer half-life for certain drugs compared to humans, which can be advantageous in drug testing, but may also present challenges in terms of accurate dosage prediction for human treatments. This extended time frame provides ample opportunity for data mining, showing potential for evaluating the efficacy and determining appropriate dosages of novel drugs for gynecological conditions (49). The findings from large animal models of gynecological inflammation, such as endometritis and pelvic inflammatory disease, have direct clinical relevance. These studies have led to the development of new therapeutic approaches, such as antibiotic and anti-inflammatory drug therapies, as well as diagnostic tools like imaging techniques that improve the detection of inflammatory lesions in the reproductive tract (50).

In recent years, experimental research on gynecological inflammation has been conducted on an ongoing basis, and the selection of animal models has been refined. However, gynecological inflammation has a multitude of potential triggers, and the selection of experimental animal models for this disease must consider not only the findings of etiological research, but also the characteristics of cyclic recurrence.

### 2.3 Infertility

Infertility represents a significant global health concern that poses a threat to women’s reproductive well-being. It is estimated that approximately 480,000 couples and 18.62 billion people of reproductive age in countries around the world are affected by infertility (51). This statistic refers to women, but similar figures can be observed for men as well. Female reproductive disorders may have a number of causes, including genetic mutations, chromosomal abnormalities, ovulatory dysfunction and genital pathologies such as those affecting the uterus (52). Approximately 25% of cases of female infertility are attributable to dysfunctions in the regulation of the hypothalamic–pituitary-gonadal axis, resulting in an endocrine hormone imbalance that affects normal ovulation, fetal development and reproductive function (53). Furthermore, it is estimated that 4–9% of women experience potential luteal phase dysfunction, which impairs endometrial receptivity, potentially leading to pregnancy difficulties and infertility (54). Further research into this disease is imperative, however, it is not possible to conduct this research directly on humans due to ethical and humanitarian considerations (55). It is therefore imperative to select an appropriate animal model for the study of this disease.

The pertinent reproductive characteristics of the large animal models are presented in tabular form in Table 3. The striking resemblance between non-human primates and humans with regard to reproductive characteristics and reproductive cycles has rendered the non-human primate model a more extensively investigated model for the study of female infertility (56). Furthermore, the model is regarded as a valuable and well-founded tool for investigating female reproductive health and disease.

**Table 3:** Fertility characteristics of large animal models.

Animal models	Gestational period (days)	Follicular phases (days)	Luteal phases (days)	Average litter size	Ovulation	Placenta type
Baboon (168)	280	—	—	1	Spontaneity	Hemoehorial placenta
Cynomolgus monkey (169)	160–170	15	13–14	1	Spontaneity	Hemoehorial placenta
Macaque monkey (162)	156–180	—	—	1–2	Spontaneity	Hemoehorial placenta
Sheep (35)	147	1–2	13–14	2–4	Spontaneity	Epitheliochorial placenta / Syndesmochorial placenta
Pig (162)	114	3–7	13–17	10	Spontaneity	Epitheliochorial placenta
Cow (170)	275–285	3–4	6–18	1	Spontaneity	Epitheliochorial placenta / Syndesmochorial placenta
Horse (171)	340	6–7	14–15	1	Spontaneity	Epitheliochorial placenta

Infertility can result from both genetic and environmental factors, including hormonal imbalances, genetic mutations, and environmental exposures (57). Large animal models, such as pigs and non-human primates, offer a unique opportunity to study these factors in a more controlled and physiologically relevant environment. For example, studies on genetically modified pigs have been used to explore the impact of specific gene mutations on ovarian function and fertility, while environmental factors such as endocrine disruptors have been investigated using rhesus monkeys (58). Therapeutic interventions, such as hormone therapy, assisted reproductive technologies (ART), and ovarian tissue transplantation, have been tested in large animal models. For instance, studies on sheep and pigs have explored the efficacy of ovarian tissue transplantation in restoring fertility in females with ovarian failure, showing promising results with successful pregnancies (59, 60). Infertility resulting from the absence or dysfunction of the uterus remains a significant challenge that has persisted as a research focus. The advent of uterus transplantation technology, involving the retrieval of a uterus from a living or brain-dead donor and its transplantation into an infertile recipient, has emerged as a promising solution. This innovative technique not only supports the healthy development of the fetus post-conception but also offers renewed optimism for individuals facing absolute uterine infertility (61). Drawing on the anatomical and physiological similarities with non-human primates, researchers have successfully transplanted autologous uteri into Cynomolgus monkey. Notably, these transplants led to natural recognition postoperatively, evidenced by three cyclic menstrual periods, culminating in the delivery of a healthy fetus via cesarean section. Conversely, baboons subjected to uterine transplantation through modified vascular anastomosis exhibited a 90% survival rate, with 60% of individuals resuming normal menstruation (62). Furthermore, the distinctive hemochorial placenta found in non-human primates facilitates direct contact between maternal blood and placental tissues, thereby extending gestation and enhancing blood nourishment in comparison to larger animal models (63). The study conducted by researchers on uterine transplantation utilizing bilateral ovarian venous reflux in pigs yielded a favorable outcome (64). The procedure resulted in successful establishment of uterine-ovarian venous reflux, facilitated by four vascular anastomoses performed with the ovarian veins. Notably, the uterine transplantation demonstrated a commendable 83% success rate in immediate reperfusion surgery (65). These findings underscore the cost-effectiveness and proficient tissue anastomosis associated with utilizing pigs in uterine transplantation techniques. Nevertheless, careful consideration is advised in experimental protocols to mitigate risks of ureteral damage.

Furthermore, sheep in large animal models are not only of moderate size, but also exhibit a high degree of similarity to women in terms of gonadal differentiation, meiosis, primordial follicle emergence, placenta development, and nutrient transport (66). Consequently, sheep are frequently employed in hormonal investigations pertaining to reproductive disorders, placental development, ovarian dynamics, and neurotransmitter assay studies. However, owing to their possession of a bicornuate uterus with a shorter length compared to humans, the arterial and venous vasculature, crucial for their blood supply, presents differences that lead to variations such as an extended thermal ischemia time during immunosuppression (67). Horses exhibit similarities to women in terms of follicular recruitment waves and the selection mechanism of dominant follicles, with their larger follicles facilitating easy observation (68). Consequently, horses have been extensively utilized in research pertaining to infertility, including instances of low ovarian reserve function. An illustrative example lies in the investigation of the heterotopic autotransplantation technique of ovarian tissues, where the utilization of horses as an experimental model demonstrated that subjecting ovarian tissues to a cooling process at 4°C for a minimum of 24 h prior to transplantation effectively preserved the quality of the tissues (69). This preservation method led to favorable rates of follicular survival and development.

Animal models utilized in infertility research must meet stringent criteria for their reproductive capabilities and should be effectively employed to mimic hormone levels, gene alterations, and other relevant factors consistent with human physiology. Large animal models have garnered significant interest in infertility studies owing to their reproductive, physiological, and anatomical advantages (70).

### 2.4 Cancer

Cancer represents a significant challenge within the biomedical field, with breast cancer emerging as the primary cause of cancer-related fatalities among women globally (71). Moreover, cervical, ovarian, and endometrial cancers, identified as the most prevalent malignant tumors, present substantial risks to women’s well-being. The complexity of malignant tumors is frequently influenced by intricate genetic and molecular signaling pathways originating from tumor cells and their surrounding microenvironment (72). Due to the limited availability of data on pathological response interactions in human subjects, experimental animals are frequently employed for *in vitro* cancer analyses. Delving deeply into cancer pathogenesis and targeted therapies can yield valuable insights for subsequent clinical trials in the field of oncology (73).

Large animal models are used to investigate the molecular mechanisms underlying gynecologic cancers, such as ovarian, cervical, and endometrial cancers. For example, research using non-human primates has provided valuable insights into the signaling pathways involved in tumorigenesis and metastasis (74). These models also allow for the study of the tumor microenvironment, which plays a crucial role in cancer progression and therapeutic response. Innovative therapeutic approaches, such as immunotherapy and gene therapy, are currently being tested using large animal models (75). For example, studies on genetically engineered pigs have been used to evaluate the efficacy of CAR-T cell therapy in treating ovarian cancer (76). Additionally, immunotherapeutic agents targeting the PD-1/PD-L1 pathway have shown promise in preclinical trials involving non-human primates (77). Farletuzumab, a newly developed inhibitor of folate receptor *α*, exhibited promising efficacy in initial trials targeting epithelial ovarian cancer (78). To investigate the toxicological profile of this compound, a series of four studies were conducted by researchers. These studies included assessments of dosage levels, tolerability, as well as repeat dosing over 28 days and 24 weeks, utilizing Cynomolgus monkeys as experimental subjects (79). The immunohistochemical analysis revealed minimal staining of Farletuzumab in the pancreatic ductal epithelium, oviductal epithelium, and pancreatic ductal epithelium of both cynomolgus monkeys and normal human tissues (80). Consequently, cynomolgus monkeys were identified as a suitable model for toxicological evaluations. Furthermore, a number of studies have indicated that the long noncoding RNA gene (long noncoding RNAs) may serve as a highly promising biomarker for various cancers (81). Among these, MIR503HG, a long-stranded non-coding RNA on the human X chromosome, has been clearly characterized in the early stages of the reproductive system, particularly in ovarian development, with down-regulation of transcripts expressed in gynecological cancers (82). Conversely, MIR503HG is currently observed to be highly conserved in only five non-human primate species. A comparison of human genes with those of selected non-human primates revealed that the exon and intron lengths of MIR503HG were well conserved in the large animal models of chimpanzees and gorillas, with the exception of the small animal model mouse lemur (83). Additionally, the uterine structure of pigs differs from that of humans. It is a bicornuate uterus, with the fetus mainly conceived in the uterine horns, and its uterine wall is relatively thin, with an average thickness of 3.88 ± 1.27 mm for the entire layer and 1.17 ± 0.15 mm for the myometrium (84). Consequently, an experiment utilizing a novel cryoablation balloon probe in a porcine model of endometrial cancer demonstrated that the effective freezing range could encompass a minimum of 2 cm in diameter, and that the procedure did not result in damage to adjacent organs (85). This successfully validated the safety and efficacy of the cryoablation technique.

Animal models have historically served as the foundation for translational research into biochemical and physiological processes, particularly in the context of cancer onset, development, and propagation in organisms (86). Furthermore, the significant heterogeneity of cancer, which differs from the human body in terms of physiology, pathology, genetics, and immunity, has resulted in an urgent need for researchers to develop animal models that can respond to the biological characteristics of humans (87). Consequently, researchers are currently engaged in the development of novel animal models utilizing innovative transgenic and transplantation technologies, which are anticipated to facilitate significant advancements in the research and development of cancer and other diseases (88).

## 3 Application characteristics of large animal models

**Figure 2:**
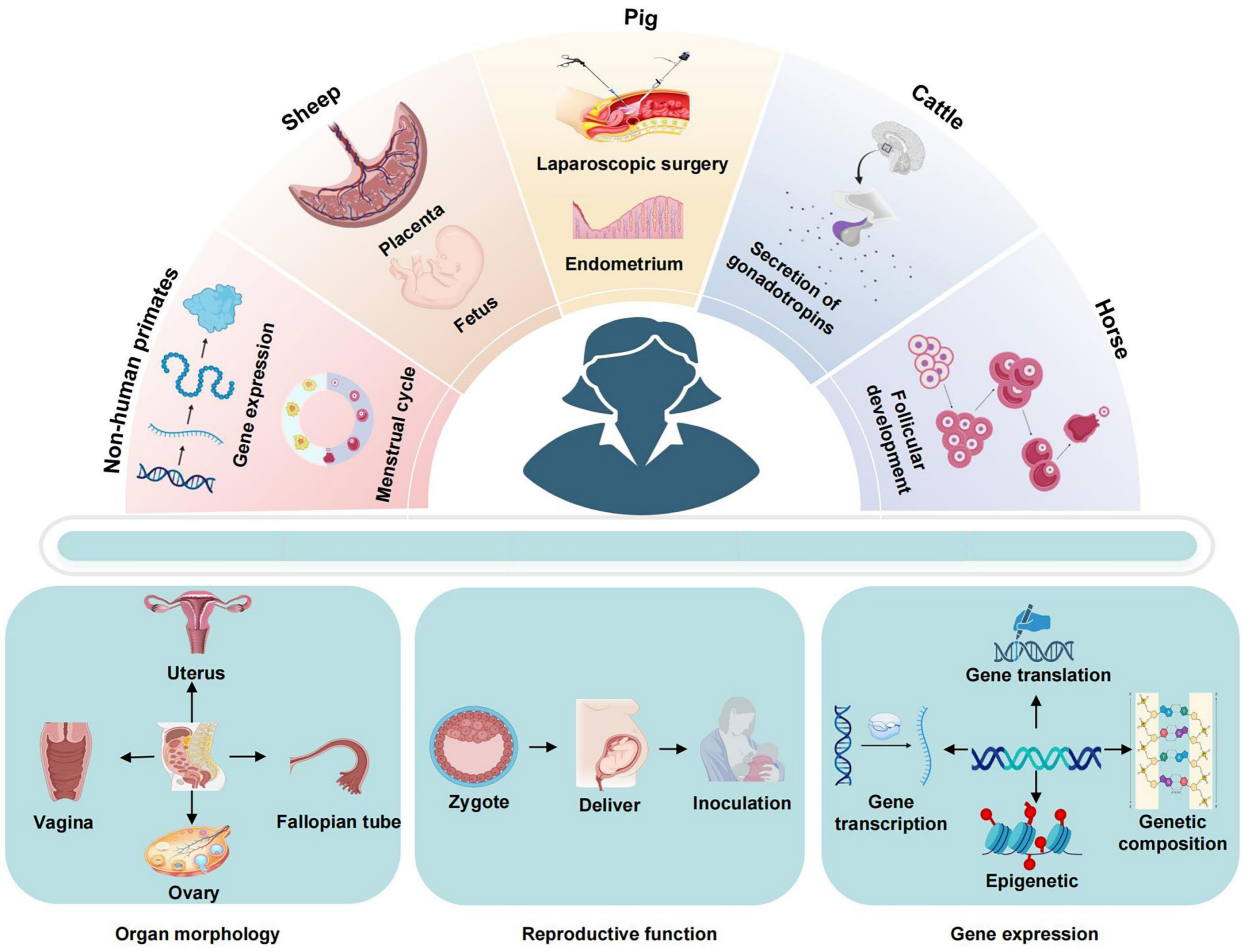
Application characteristics of large animal models.

The research of large animal models in the field of gynecology is primarily concerned with the elucidation of disease mechanisms and the advancement of diagnostic and therapeutic techniques. As medical research progresses and science and technology advance, the creation of experimental models is becoming increasingly sophisticated, enabling more accurate and efficient simulation of human disease states, as illustrated in Figure 2 and Table 4. This provides a crucial experimental foundation for investigating disease pathogenesis, developing diagnostic methods and advancing therapeutic measures.

**Table 4:** Comparison of small animal models and large animal models.

Aspect	Small animal models	Large animal models
Physiological similarity	Limited similarity to humans	Closer resemblance to human physiology
Anatomical structure	Simplified pelvic cavity, limited reproductive organ complexity	Complex pelvic anatomy, more representative of human structure
Gene expression	Significant genetic differences with humans	More similar to human gene expression patterns
Cost	Low cost, easy to maintain	High cost, maintenance-intensive
Ethical Considerations	Fewer ethical concerns, but still present	Higher ethical concerns, especially with primates
Use in Disease Modeling	Suitable for basic research, less applicable for complex diseases	Better for studying complex diseases, drug efficacy, and organ transplantation

The non-human primate model stands out among large animal models in gynecology for its strong resemblance to humans. Non-human primates exhibit similarities with humans in terms of neuroendocrine regulation of the reproductive system, as well as in circulatory functions of the ovaries and reproductive tract critical for establishing and managing the maternal-fetal-placental connection during pregnancy (89). The most commonly used non-human primate models in laboratory settings are macaques and cynomolgus monkeys, which exhibit approximately 95% genetic homology to humans. Additionally, baboons are regarded as the most suitable models for studying embryonic stem cells and artificial reproductive technology (ART) (90). This makes non-human primates a valuable resource for investigating female infertility, facilitating advancements in uterine transplantation techniques, minimizing damage from organ ischemia and reperfusion, and enhancing postoperative outcomes (91). Furthermore, non-human primates can replicate infertility scenarios such as placental dysplasia and maternal-fetal dysfunction resulting from environmental influences. As such, they are frequently employed in studies related to infertility treatment, hormonal disorders analysis, pharmacokinetics of pregnancy-related compounds, and antiretroviral therapy.

The pig, alongside non-human primates, possesses a unique uterine structure characterized by a bicornuate uterus with prominent lumen uterine horns positioned adjacent to the bladder (92). These uterine horns are deemed suitable for simulating laparoscopic ureteral reconstruction due to their sufficient length, offering an alternative to dilated ureters. Furthermore, the stromal and perivascular regions of the porcine uterus contain marker proteins for mesenchymal stem cells (MSCs), serving as potential sites for stem/progenitor cell residence. Notably, the presence of unevenly distributed stromal CD105 + and CD106 + cells suggests their role as precursors for peripheral vascular growth (93). This similarity in the endometrial MSC phenotype between pigs and humans underscores the utility of pigs as a valuable large animal model for investigating endometrial function in translational research (94). Additionally, pigs are frequently utilized as models for training and experimental purposes in various pelvic surgical procedures. Junior surgeons often practice laparoscopic surgery on pig models using advanced 3D technology, while pigs are also employed as test subjects for suturing techniques in gynecology residency training programs (95).

Sheep serve as vital models in the examination of fetal physiology throughout pregnancy. They are ideal for translational research questions due to their gestational length, the availability of a single fetus for catheter insertion, and the similarity in fetal size to humans, which facilitates delivery using standard clinical techniques (96). Furthermore, fetal sheep preparations provide an effective means of treating preterm labor associated with ischemia-hypoxia or maternal-fetal infections, as evidenced by near-full-term fetal sheep in preclinical studies (97). The use of instrumented fetal sheep in utero presents numerous practical benefits compared to extrauterine models featuring cerebral gyration. The extended gestation period of fetal sheep allows for the strategic selection of an appropriate estrous phase for the induction and evaluation of brain injury, highlighting the susceptibility of preterm fetal sheep to acute and chronic brain damage resembling that observed in humans in terms of histopathological traits.

The utilization of large animal models presents an opportunity to enhance our comprehension of ovarian function, specifically in cattle and horses, due to their possession of sizable antral follicles and growth stages akin to the dominant ovulatory follicle in humans (98). As a result, cattle and horses serve as appropriate subjects for investigating follicle dynamics through the application of ultrasonography. It has been demonstrated that the temporal events that occur in cattle during the menstrual cycle are consistent with findings in women (99). The decline in maternal fertility with age is associated with the initiation of follicular waves, leading to a 4-day cyclic increase in gonadotropin levels (100). The circulating concentrations of gonadotropins are increased in the late reproductive stages, which is accompanied by a decrease in inhibin B secretion (101). Furthermore, the antral follicle count (AFC) has been employed as a marker of reproductive potential and a predictor of ovarian response to hyperstimulation (102). As such, investigations focusing on the depletion of primordial follicles, diminished antral follicle reserve, changes in hormone secretion, and meiotic abnormalities leading to female infertility may benefit from mechanistic exploration using cattle as an animal model (103).

## 4 Conclusion

As a crucial instrument in translational research within the context of clinical trials for human diseases, the suitability of the animal model to the disease under investigation hinges on the efficacy of the experimental process and the scientific rigor of the resulting data.

### 4.1 Comparison of differences in gynecological research between large and small animal models

Animal models have emerged as a valuable asset in gynecological research, particularly in light of ethical considerations within the field. Small animal models, notably rodents, are prevalent due to their accessibility and cost-effectiveness. Nonetheless, a comprehensive understanding of gynecological diseases necessitates a thorough characterization of the phenotype and genotype of experimental animals (104). Despite the fact that humans and rodents share a number of fundamental biological processes, the differences between the two species become increasingly apparent as research progresses (105). In terms of anatomical structure, there are significant differences between small animal models and humans, particularly in the pelvic structure and reproductive organs, which exhibit notable species-specific variations (106). Small animals have a relatively uncomplicated pelvic cavity primarily dedicated to housing the reproductive organs. In contrast, the female human pelvis not only accommodates the reproductive organs but also interfaces with the rectum, bladder, and other vital organs, directly influencing the birthing process (107). Additionally, the commonly used ‘Y’-shaped uterus in female mice differs substantially from the inverted ‘pear-shaped’ uterus in humans, highlighting the inadequacy of small animal models in replicating the reproductive system’s morphology (108). With regard to physiological function, no small animal models have been identified to exhibit natural menstruation through cyclic endometrial interstitial metamorphosis, with the exception of the spiny mouse that has been recently discovered. The induction of exogenous hormones or the removal of the ovaries does not fully replicate the cyclical hormonal fluctuations and blood supply to the spiral arteries observed during normal menstruation (109). Conversely, the spiny mouse is distinguished as the sole rodent species to naturally undergo menstruation. Being a native North American species, it is susceptible to environmental factors, and its utility in menstrual research is constrained by its distinct physiology and lack of immune response to antigens and antibodies (110). With regard to gene expression, the utilization of transgenic mice in investigating gynecological malignancies has seen extensive adoption in gene expression studies (111). In addition to their low cost of application, their diverse immunodeficiency strains can be employed in the experimental development of cancer drugs (112). Nonetheless, comparative analysis of mouse and human genes has revealed substantial variances in transcription factor binding sites, ranging from 41 to 89% of instances (113). Such disparities have impeded investigations into the genetic, immunological, and molecular underpinnings of gynecological disorders in murine models.

### 4.2 Opportunities and challenges faced by large animal models

It is evident that there is a paucity of suitable models for the study of diseases in the field of gynecology. The discrepancies between small animal models and women in terms of anatomy, organ morphology, reproductive function and gene expression have led to a growing interest in the study of large animal models. Large animal models exhibit greater physiological similarity with humans in terms of genetics, structure and function, which renders their experimental results more informative (114). Moreover, due to the closer proximity in body size to humans, large animal models manifest immune and metabolic responses that more closely mirror those in humans (115). As a result, the experimental data derived from large animal models are deemed more dependable and enable more accurate predictions in assessing the safety and effectiveness of drugs for gynecological conditions. In the current era, the advent of genome editing technology (CRISPR-Cas9 system) has propelled the advancement of cellular imaging, gene expression regulation, epigenetic modification, therapeutic drug development, functional gene screening, and genetic diagnosis (116). Non-human primates have emerged as viable translation targets due to their analogous capability to humans in gene editing and expression, showcasing commendable gene modification and delivery efficiency (117). In the field of biomedical research, cloning has also been employed in the replication of genes and the development of immunological agents in large animal models (118). Notably, cloned pigs exhibit genetic sequences more akin to humans, allowing for advances in clinical research on human embryonic development through the selection of cloned pigs for embryo biopsy in conjunction with microproteomics (119). In recent years, the development of molecular biology has also facilitated the rapid advancement of multifunctional stem cell technology (120). Nevertheless, the lack of *in vivo* data on human development makes it challenging to ascertain the extent to which *in vitro* models accurately reflect *in vivo* processes (16). While primate hematopoietic stem cells share an origin with pre-implantation epiblasts, their transcriptomic profile aligns more closely with post-implantation epiblasts than with traditional rodent models (121). Consequently, to address potential artifacts and species discrepancies and gain deeper insights into human development, an integrated approach encompassing analyses across various platforms (*in vivo, ex vivo, in vitro*) and species (hominids, primates) is warranted (122). Furthermore, the integration of organ bioengineering with the use of large animal models has significantly advanced the field of organ replacement in the context of gynecological disorders (123). Research endeavors focusing on uterine transplantation have been conducted in various large animal species such as pigs, sheep, and non-human primates. This represents a significant advancement in the field of bioengineering of the female reproductive system.

In the realm of gynecological research, the utilization of large animal models serves to better replicate the physiological and pathological manifestations of human gynecological diseases, offering researchers a more authentic experimental setting akin to clinical scenarios. This not only enhances the realism of the studies conducted but also furnishes a crucial foundation for the development and evaluation of pharmaceutical interventions (124). Nevertheless, the exploration of large animal models necessitates comprehensive data support through the acquisition of substantial sample sizes (125). Consequently, it becomes imperative to both optimize cost-effectiveness to mitigate financial constraints and bolster the technical proficiency of personnel in order to elevate the scientific rigor and safety standards of experimental endeavors (126). Ethical considerations pose significant challenges when implementing large animal models for experimental research, particularly in the case of non-human primate models like chimpanzees, which share a closer genetic and evolutionary relationship with humans (127). The utilization of non-human primates in research has sparked intense debates, particularly following the 2015 legislation in the United States that designated captive chimpanzees, including those in laboratory settings, as endangered species (128). This development underscored the necessity for stringent ethical scrutiny in experimental research involving non-human primates, aligning with elevated ethical standards (129). In contrast to countries like the United Kingdom and the United States, Brazil engages in comparatively less research involving non-human primates (130). Nonetheless, Brazil’s National Commission for the Control of Experiments on Animals (CONCEA) has been consistently revising its ethical guidelines pertaining to primate research (131). Notably, the UK Parliament has been a trailblazer in enacting legislation concerning animal usage, with the principles of the 3Rs—Reduce, Replace, and Improve—originating from the UK and now serving as a guiding framework for animal research worldwide. It is evident that ethical and transparent practices are imperative in experimental research involving large animal models to ensure the ethical justification of animal use, particularly in instances of invasive procedures and reproductive studies, and to uphold the strict adherence to animal protection regulations safeguarding the welfare and rights of animals (131).

### 4.3 The relationship between large animal models and new alternative models

Preclinical large animal models are integral to drug development as they replicate the pharmacokinetic characteristics of the human body, facilitating comprehension of drug absorption, distribution, metabolism, and excretion processes (132). These models establish a foundation for determining appropriate drug doses and dosing schedules, assessing a drug’s therapeutic effectiveness against specific diseases through disease modeling and therapeutic trials, and furnishing data that supports drug marketing efforts. Nonetheless, the utility of large animal models in drug safety evaluations and toxicology studies is constrained by their typically high costs, ethical considerations, unsuitability for high-throughput bioassays and extensive drug screenings, and limited ability to faithfully mirror human responses (133). Consequently, there is a growing imperative to develop preclinical drug testing models with enhanced physiological relevance to more accurately replicate complex human-related conditions and thereby enhance the efficacy of clinical trials.

The emergence of the ‘organ-on-a-chip’ concept in recent years presents a promising avenue for modeling human physiology *in vitro*. This innovative approach involves replicating the physiological conditions and functions of human organs within a microfluidic chip platform (134). By incorporating strategies for co-culturing with microorganisms and utilizing multi-parameter control approaches, researchers have been able to create a more sophisticated *in vitro* model (135). The ‘organ-on-a-chip’ technology has been suggested as a viable replacement for animal studies and as a method to address the underrepresentation of female participants in human clinical trials (136). However, existing single-organ microarrays lack systemic dimensions and inter-organ communication (137). To enhance the efficacy of drug development, integrating large animal models with organ-on-a-chip technology has been proposed. By combining preliminary drug screening in large animal models with comprehensive in vitro experiments using organ-on-a-chip systems, researchers can achieve more accurate predictions of drug responses and effects in the human body. Furthermore, AI has shown considerable benefits in various medical domains, including disease diagnosis, precision medicine, and drug development (138). The utilization of AI technology enables the rapid and direct transformation of vast quantities of medical data, such as medical images and gene expression, into actionable experimental parameters. This integration enhances the efficiency of drug development processes, eliminates human intervention and subjective biases, and significantly enhances the accuracy of data analysis (139). By leveraging AI technology in conjunction with large animal models to collect and analyze data from drug experiments and disease simulations, insights into disease progression patterns and drug response kinetics can be expedited (140). Furthermore, the combined application of AI and medical imaging technologies, such as ultrasound, MRI, and CT scans, allows for comprehensive imaging and data analysis of large animal models, thereby enhancing the precision and predictive capabilities of disease diagnostics (141). Additionally, leveraging the genetic background, physiological traits, and other pertinent data of large animal models, personalized diagnosis and treatment strategies can be formulated through the integration of AI technology.

In brief, the significance of employing large animal models in gynecological research is unequivocal, as evidenced by their substantial utility in advancing women’s health. In the forthcoming era, these large animal models are poised to leverage their inherent strengths in conjunction with innovative technologies, thereby catalyzing transformative breakthroughs in medical research and fostering accelerated growth and advancement in the field.

### 4.4 Ethical considerations and the 3Rs principle in gynecological research

The use of large animal models in gynecological research offers significant scientific advantages, particularly for studying complex reproductive processes. However, it also raises critical ethical concerns, especially regarding the use of non-human primates (NHPs) (16). Recent legislative developments, such as the European Union’s proposed ban on NHP research by 2025, highlight the growing ethical scrutiny surrounding animal research (142). This policy shift underscores the need for alternative approaches that reduce reliance on primates in biomedical studies (143).

The 3Rs principle (Replacement, Reduction, Refinement) provides a framework to address these ethical concerns (144). Replacement encourages the use of alternative models, such as organ-on-a-chip systems and AI-based simulations, which can replicate human physiological processes without the need for animal testing (145). These technologies are particularly promising in gynecological research, offering ethical and scientifically valid alternatives to traditional animal models.

Reduction emphasizes minimizing the number of animals used in experiments by refining study designs and utilizing more efficient models. Large animal models, due to their closer physiological similarity to humans, can reduce the number of animals required in research by providing more relevant data in fewer subjects. Refinement focuses on improving research methodologies to minimize animal suffering, such as using non-invasive imaging techniques like MRI and ultrasound, which allow for detailed observations of reproductive processes without the need for invasive procedures (146).

In conclusion, while large animal models remain essential for advancing gynecological research, it is imperative that ethical considerations are prioritized. By adhering to the 3Rs principle, researchers can ensure that scientific progress is made responsibly, with a continued focus on reducing animal use and improving welfare standards.
